# Clinical analysis of 314 patients with high-grade squamous intraepithelial lesion who underwent total hysterectomy directly: a multi-center, retrospective cohort study

**DOI:** 10.1186/s12885-024-12342-2

**Published:** 2024-05-09

**Authors:** Lingyu Lei, Lin Zhang, Yahong Zheng, Wei Ma, Fang Liu, Dongmei Li, Keming Chen, Yong Zeng

**Affiliations:** 1 Department of Obstetrics and Gynecology, The First Affiliated Hospital of Yangtze University, 8 Hangkong Road, Shashi District, Jingzhou, Hubei China; 2 Department of Obstetrics and Gynecology, Jianli County People’s Hospital, Jingzhou City, Hubei Province China; 3 Department of Obstetrics and Gynecology, Shishou People’s Hospital, Mingzhu Street, Shishou, Hubei China

**Keywords:** HSIL, Total hysterectomy, Pathological upgrading, Occult cervical cancer

## Abstract

**Objective:**

To identify the risk factors of cervical high-grade squamous intraepithelial lesion(HSIL) complicated with occult cervical cancer and standardize the management of initial treatment for HSIL.

**Method:**

The clinical data of patients who underwent total hysterectomy directly due to HSIL in the obstetrics and gynecology department of two tertiary hospitals and three secondary hospitals from 2018 to 2023 were collected. Their general characteristics, pathological parameters and survival status were analyzed. Logistic regression model was used to analyze the correlation between clinical parameters and postoperative pathological upgrading.

**Result:**

1. Among the 314 patients with HSIL who underwent total hysterectomy directly, 73.2% were from primary hospitals. 2. 25 patients (7.9%) were pathologically upgraded to cervical cancer, all of which were early invasive cancer. 3. Up to now, there was no recurrence or death in the 25 patients with early-stage invasive cancer, and the median follow-up period was 21 months(range 2–59 months). 4. Glandular involvement(OR 3.968; 95%CI 1.244–12.662) and lesion range ≥ 3 quadrants (OR 6.527; 95% CI 1.78–23.931), HPV 16/18 infection (OR 5.382; 95%CI 1.947–14.872), TCT ≥ ASC-H (OR 4.719; 95%CI 1.892–11.766) were independent risk factors that affected the upgrading of postoperative pathology. 5. The area under the curve (AUC) calculated by the Logistic regression model was 0.840, indicating that the predictive value was good.

**Conclusion:**

There is a risk of occult cervical cancer in patients with HSIL. Glandular involvement, Lesion range ≥ 3 quadrants, HPV 16/18 infection and TCT ≥ ASC-H are independent risk factors for HSIL combined with occult cervical cancer. The prognosis of biopsy-proved HSIL patients who underwent extrafascial hysterectomy and unexpected early invasive cancer was later identified on specimen may be good.

**Supplementary Information:**

The online version contains supplementary material available at 10.1186/s12885-024-12342-2.

## Introduction

Cervical High-grade Squamous Intraepithelial Lesion(HSIL) is a precancerous lesion of cervical cancer. McCredie found that if HSIL is not treated, approximately 31% to 50% of patients will progress to cancer within 30 years, and with conventional treatment, the risk will be reduced to 0.7% [1]. According to the guidelines of the American Society for Colposcopy Pathology (ASCCP) (2019) [2] and the consensus of China experts [3], the patients diagnosed with HSIL by histopathology can be treated with cervical resection. The purpose of treatment is to completely remove the lesions and prevent cancer. At the same time, it can be further diagnosis to avoid missed diagnosis of early or occult cervical cancer. Therefore, total hysterectomy directly is not recommended as the routine first-choice treatment for HSIL.

As a developing country, China has an uneven distribution of medical resources in different regions, and the ability of doctors is also uneven. In addition, it is difficult to distinguish the boundary between cervical and vaginal fornix due to obvious cervical atrophy and fornix disappearance after menopause, and the cervix cannot be completely exposed due to vaginal contracture [3, 4]. These conditions increase the difficulty of conization surgery(CKC(cold knife conzation) and LEEP(loop electrosurgical excision procedure), which leads to the phenomenon of blindly expanding the indications of hysterectomy, especially in primary hospitals. The direct hysterectomy for HSIL may lead to overtreatment on the one hand, and may lead to undertreatment if the postoperative pathology upgrades on the other hand [3, 5]. Total hysterectomy may also lead to a series of complications such as ureteral, bladder and nerve injury. Studies found among the patients undergoing extrafascial hysterectomy due to cervical intraepithelial neoplasia III (CINIII), the rate of upgrading to invasive carcinoma was 10.38**–**17% [5–7]. Therefore, it is particularly important to standardize the diagnosis and treatment of the initial treatment of HSIL. This study will retrospectively analyze the clinical data of patients with HSIL undergoing total hysterectomy directly and summarize the risk factors of postoperative pathological upgrading, in order to guide clinical diagnosis and treatment.

## Research methods and data analysis

### Data collection

Clinicopathological data of patients undergoing total hysterectomy directly for HSIL in gynecology and obstetrics departments of Jingzhou First People’s Hospital, Jingzhou Central Hospital, Shishou People’s Hospital, Luoyang Hospital and Jianli County People’s Hospital from 2018 to 2023 were collected (This study has been approved by the Ethics Committee and informed consent was obtained from all patients and/or their families). The inclusion criteria of this study were as follows: (1) Preoperative colposcopy was performed to evaluate the nature and extent of cervical lesions, and the cervical histopathological results were HSIL; (2) No history of HSIL / HSIL+ diagnosis and surgical treatment; (3) Direct hysterectomy was performed for initial treatment; (4) Provide approximately complete clinical data. The exclusion criteria as follows: Thin-prep cytology test (TCT) results were cytology squamous cell carcinoma or adenocarcinoma.

For human papillomavirus (HPV) testing and genotyping, the Cobas HPV test, which is based on a real-time polymerase chain reaction (PCR) system, was performed (Cobas^®^ 4800; Roche Molecular Diagnostics). This method can detect 18 high-risk HPV (HR-HPV) types. Multiple HR-HPV infections were defined as two or more HR-HPV infections.

Thin-prep cytology test (TCT): Cervical epithelial cells were collected using a cervical brush and diagnosed by a cytologist according to the TBS (2001) system. TCT results include: No Intraepithelial Lesion or Malignancy (NILM); atypical squamous cell of undefined significance (ASC-US); low-grade cervical intraepithelial lesion (LSIL); atypical squamous cells cannot exclude high-grade squamous intraepithelial lesion (ASC-H); high-grade cervical intraepithelial lesion (HSIL); and atypical glandular cells (AGC). TCT ≥ ASC-H is defined as ASC-H, HSIL and AGC.

Colposcopy Procedure:Refer to the terms recommended by International Federation for Cervical Pathology and Colposcopy(IFCPC) 2011 for colposcopy. Acetic acid test was carried out during the examination, and the cervix and the fornix were completely covered with 3~5% acetic acid cotton balls and wet applied for 60 s. The changes of cervix and vaginal epithelium were examined systematically from low power to high power lens and the types of transformation zone(TZ) were determined. The transformation zone of type II and III (TZ II/III) could be observed with cervical tube dilators. For patients with inadequate colposcopy, especially those with TZ III, a Endocervical curettage(ECC) is performed (except during pregnancy). Acetic acid can be reused if necessary. Second, compound iodine test can be used on the basis of acetic acid test (with a history of iodine allergy prohibited). The primitive squamous epithelium and mature metaplasia epithelium after puberty were rich in glycogen and were brown after iodine staining. In postmenopausal or estrogenic squamous epithelium, immature metaplasia squamous epithelium, columnar epithelium, intraepithelial lesions, cancer and inflammatory lesions, the epithelium was not colored or showed varying degrees of yellow after iodine staining. A colposcopically guided biopsy of the lesion site in the cervical (or vaginal) region describes the size of the lesion as the number of cervical quadrants covered by the lesion or the size of the percentage of the cervix surrounded by the lesion. The maximum tissue diameter of each “bite test” should be no less than 3 mm. The specimens should be labeled and packaged separately, and fixed in 4% neutral formaldehyde solution before being sent for pathological examination.

Lesion range: The cervix is divided into four quadrants, with each quadrant accounting for 25%. Based on the colposcopically guided biopsy results, the percentage of each case in the external region of the cervix was specifically assessed to determine the final scope of lesions. Lesion range ≥ 3 quadrants means that lesion involvement reaches 3 or more quadrants.

According to the standardized nomenclature scheme of cervical lesions jointly issued by the College of American Pathology (CAP) and the American Society of Colposcopy and Cervical Pathology (ASCCP) in 2012, the cervical intraepithelial lesion was divided into HSIL and LSIL. LSIL includes HPV/CIN I; HSIL includes CIN II and III [8]. We reclassified patients with cervical intraepithelial lesions according to this protocol. Patients with postoperative invasive carcinoma are considered to have advanced pathology.

### Statistical methods

SPSS26.0 software was used for statistical analysis. Count data were expressed as absolute numbers and percentages (%), and X^2^ test or Fisher’s exact test was used. The measurement data conforming to normal distribution after normality test were expressed as mean ± standard deviation and analyzed by t test. Data that do not conform to the normal distribution were expressed as median (quartile) and analyzed by rank sum test. Logistic regression model was used to analyze the independent risk factors affecting the upgrading of postoperative pathological examination results to cervical cancer. The predictive value was evaluated by the area under the receiver operating characteristic (ROC) curve (AUC) calculated by the logistic regression model. An AUC of 0.9–1 indicates that the predictive value of this indicator is very high. An AUC of 0.7–0.9 indicates good predictive value. An AUC of 0.5–0.7 indicates average predictive value. And an AUC < 0.5 indicates no predictive value. *P* value < 0.05 was considered statistically significant.

## Result

A total of 314 patients were enrolled in the study, including 84(26.8%) in tertiary hospitals and 230 (73.2%) in secondary hospitals. The mean age was 59.7 years, the median age was 59 years (range 40–84 years), and 270 patients were postmenopausal. The families with low education and low income accounted for 88.5% and 77.1%, respectively. 225 cases had ≥ 3 pregnancies; 256 cases had ≥ 2 births; 6 cases had smoking history; 22 cases had immune system diseases(include: Rheumatoid Arthritis, Diabetes, Thyroid disease and so on); 58 cases with other indications of gynecological surgery. Preoperative colposcopy showed that 198 cases (63.1%) had TZ III, 185 cases (58.9%) had lesion range ≥ 3 quadrants, and histopathologic biopsy 177 cases (56.4%) had glandular involvement. Among the 103 patients (32.8%) visiting the hospital due to symptoms, 71 (22.6%) had vaginal bleeding, 23 (7.3%) had abnormal vaginal secretions, and the remaining 9 (2.9%) presented with abdominal pain, discomfort in the lower abdomen or vulva. The vast majority of patients (93.0%) had HPV infection, including 285 patients (90.8%) HR-HPV positive, 130 patients (41.4%) HPV16/18 positive and 139 patients(44.3%) multiple HR-HPV positive. Only 22 patients (7.0%) were HPV negative. TCT showed abnormal results in 217 cases (69.4%), including 35 cases LSIL (11.1%), 117 cases ASC-US (37.3%), 34 cases HSIL (10.8%), 29 cases ASC-H (9.2%), and 2 cases AGC (0.6%), 1 case (0.3%) of abnormal TCT but unknown results. A total of 65 patients had TCT ≥ ASC-H. In addition, we only collected the squamous cell carcinoma antigen(SCCA) results of 89 patients and pelvic MRI results of 38 patients, including 20(22.5%) patients with elevated SCCA results and 27 (71.1%) patients with abnormal pelvic MRI results (Tables 1 and 2).

**Table 1:** Demographics and clinical characteristics of study population

Parameter	Number
Age	
≥ 60	155
50–60	133
40–50	26
Region	
City	72
Rural	242
Educational level	
≥ High school	36
High school	278
TZ	
1	15
2	101
3	198
HPV infection	
16/18	130
HR-HPV	285
Multiple HR-HPV	139
Negative	22
TCT	
LSIL	35
ASC-US	117
HSIL	34
ASC-H	29
AGC	2
MRI	
Normal	11
Abnormal	27

**Table 2:** Univariate analysis of risk factors for pathological upgrading after total hysterectomy directly for HSIL

Parameter	Number		OR (95%CI)	*P*-values
	Upgrade (%)	Aggregate		
Age				
≥ 60	15 (9.7%)	155	1.596 (0.694–3.671)	*P* = 0.271
< 60	10 (6.2%)	159		
Postmenopausal				
Yes	23 (8.5%)	270	1.955 (0.444–8.603)	*P* = 1.955
No	2 (4.5%)	44		
Gravidity				
≥ 3	21 (9.3%)	225	2.187 (0.729–6.564)	*P* = 0.163
< 3	4 (4.4%)	89		
Parity				
≥ 2	20 (7.8%)	256	0.898 (0.323–2.502)	*P* = 0.837
< 2	5 (8.6%)	58		
Smoking				
Yes	0	6	0.979 (0.963–0.996)	*P* = 0.467
No	25 (8.1%)	308		
Immune system diseases				
Yes	0	22	1.055 (0.233–4.772)	*P* = 0.944
No	25 (8.6%)	292		
TZIII				
Yes	19 (9.6%)	198	1.946 (0.754–5.022)	*P* = 0.169
No	6 (5.2%)	116		
Glandular involvement				
Yes	21 (11.9%)	177	4.476 (1.499–13.366)	*P* = 0.007
No	4 (2.9%)	137		
Lesion range ≥ 3 quadrants				
Yes	22 (11.9%)	185	5.669 (1.659–19.364)	*P* = 0.006
No	3 (2.3%)	129		
HPV 16/18 infection				
Yes	19 (14.6%)	130	4.992 (1.934–12.885)	*P* = 0.001
No	6 (3.3%)	184		
Multiple HR-HPV infection				
Yes	12 (8.6%)	139	1.148 (0.506–2.604)	*P* = 0.74
No	13 (7.6%)	171		
TCT ≥ ASC-H				
Yes	14 (21.5%)	65	5.939 (2.55–13.835)	*P* < 0.001
No	11 (4.4%)	248		
Symptoms				
Yes	12 (11.4%)	105	1.945 (0.855–4.428)	*P* = 0.113
No	13 (6.2%)	209		
SCCA				
Upgrade	5 (25%)	20	1.967 (0.584–6.621)	*P* = 0.269
Normal	10 (14.5%)	69		

A total of 25 cases (9 cases of stage IA1, 6 cases of stage IA2, and 10 cases of stage IB1) had upgraded pathological results after surgery, with an average age of 63 years and a median age of 62 years (range 43–77 years). Most of them come from low education (92%) and low income (68%) families. 5 cases were combined with other indications of gynecological surgery. HPV screening results showed that 19 cases (76%) were HPV16/18 positive and 12 cases(48%) were multiple HR-HPV positive. TCT results showed that there were 1 case NILM (0.04%), 10 cases ASC-US (40%), 6 cases ASC-H (0.24%), 7 cases HSIL (0.28%), and 1 case AGC (0.04%). SCCA increased in 5 patients (20%). Pelvic MRI was performed in 8 patients, all from tertiary hospitals, and the results were abnormal in 5 patients(3 cases of cervical canal thickening and 2 cases of uneven cervical mucosa texture). 36% of patients came to hospital with related symptoms. The primary surgical procedure for all patients was extrafascial hysterectomy directly. Among the 13 patients from tertiary hospitals, 11 patients underwent intraoperative frozen section, and 8 patients were found to have malignant tumors, and then pelvic lymph node dissection was performed by expanding the scope of surgery. A total of 8 patients (61.5%) received adjuvant therapy(3 cases with radiotherapy, 3 cases with chemotherapy, 2 cases with radiotherapy plus chemotherapy). None of the 12 patients from secondary hospitals underwent rapid pathologic examination during operation, and no adjuvant treatment was given after operation. Up to now, we have found no recurrence or death cases, and the median follow-up period was 21 months (range 2–59 months) (Attached Table).

We analyzed the correlation between the clinical data of the patients and the pathological upgrading after hysterectomy. Univariate analysis showed that Age (*P* = 0.271), Postmenopause (*P* = 0.375), Gravidity (*P* = 0.163), Parity (*P* = 0.837), Smoking (*P* = 0.467), Immune system diseases (*P* = 0.944), TZ III (*P* = 0.169), Multiple HR-HPV infection (*P* = 0.720), Symptoms (*P* = 0.113) and SCC (*P* = 0.269) were not associated with pathological upgrade to cervical cancer after total hysterectomy directly. However, Glandular involvement (*P* = 0.007), Lesion range ≥ 3 quadrants (*P* = 0.006), HPV 16/18 infection (*P* = 0.001), and TCT ≥ ASC-H (*P* < 0.001) were associated with postoperative pathological upgrading (Table 2).

Feasible variables that were significantly associated in univariate analysis were entered into the logistic multivariate regression equation. Logistic regression analysis showed that Glandular involvement (OR 3.968; 95%CI 1.244–12.662; *P* = 0.02), Lesion range ≥ 3 quadrants (OR 6.527; 95% CI 1.78–23.931; *P* = 0.005), HPV 16/18 infection (OR 5.382; 95%CI 1.947–14.872; *P* = 0.001), TCT ≥ ASC-H (OR 4.719; 95%CI 1.892–11.766; *P* = 0.001) were independent risk factors for the upgrade of postoperative pathological results (Table 3).

**Table 3:** Multivariate analysis of risk factors for pathological upgrading after total hysterectomy directly for HSIL

	B	S.E.	Wald	Sig.	OR (95%CI)		
Glandular involvement	1.378	0.592	5.42	0.02	3.968	1.244	12.662
Lesion range ≥ 3 quadrants	1.876	0.663	8.008	0.005	6.527	1.78	23.931
HPV 16/18 infection	1.683	0.519	10.53	0.001	5.382	1.947	14.872
TCT ≥ ASCH	1.552	0.466	11.077	0.001	4.719	1.892	11.766

**Fig. 1:**
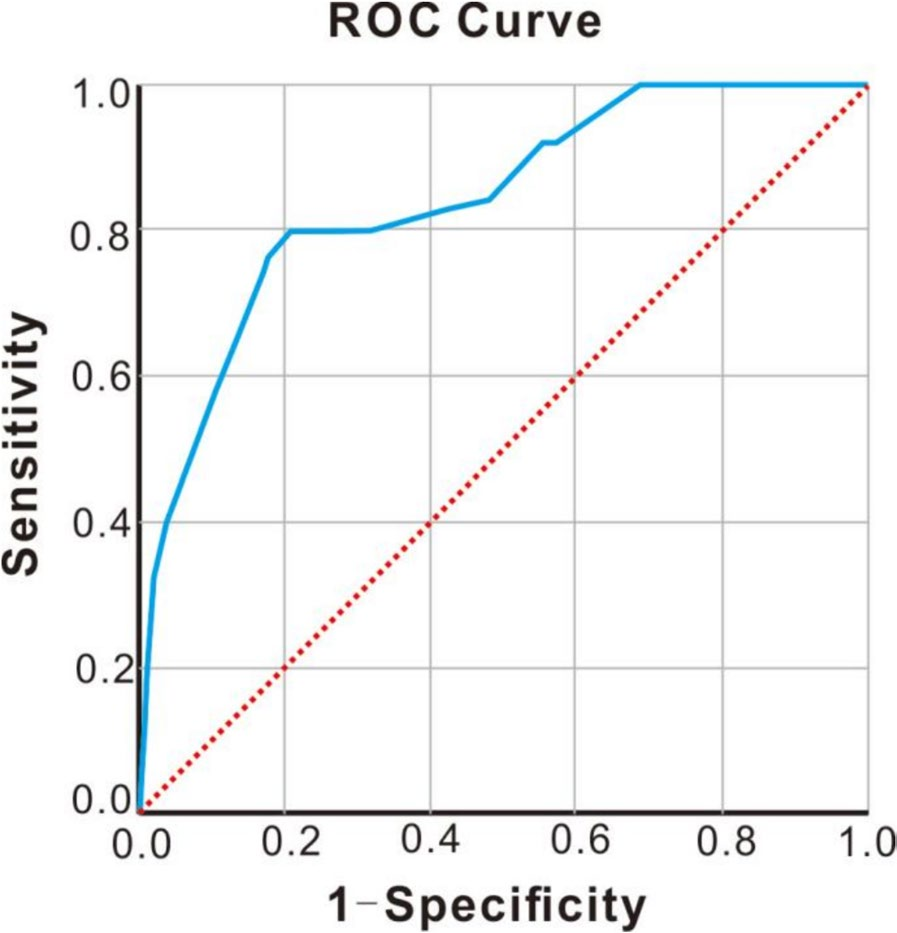
ROC prediction curve for pathological upgrading after total hysterectomy for HSIL

Independent risk factors for postoperative pathological upgrading in multivariate analysis were included in the logistic regression model, and the ROC curve of the model was plotted to evaluate its predictive value. The AUC calculated by the Logistic regression model was 0.84 (95%CI:0.758–0.922), Specificity 0.79, Sensitivity 0.8, indicating its good predictive value (Fig. 1).

## Discussion

It has been reported that HSIL can coexist with cervical cancer and the risk of cancer increases with age [9, 10]. If patients undergo total hysterectomy directly, they may face the risk of upgrading postoperative pathological results. The high risk factors of HSIL with cervical invasive cancer include postmenopausal, lesions involved glands, lesion range ≥ 2 quadrants, lesions involving cervical canal [11]. In this study, we found that the rate of pathological upgrading after surgery was 7.9%, which is similar to earlier reported in the literature [5–7]. However, Through univariate and multivariate logistic regression analysis, we found that Glandular involvement, lesion range ≥ 3 quadrants, HPV 16/18 infection and TCT ≥ ASC-H were independent risk factors for postoperative pathological upgrading, which was slightly different from previous reports [11, 12]. In addition, ROC curve analysis was performed to explore their predictive value, and ROC curve results showed that they had a good value in predicting postoperative pathological upgrading (AUC area under the curve was 0.84).

There is no consensus on the correlation between glandular involvement and postoperative pathological upgrading. This study found that glandular involvement was an independent risk factor for postoperative pathological upgrading. Deng Lei believes that involvement of glands is related to the occurrence of invasive cervical cancer, possibly because atypical proliferating cells are concealed in normal cells and progress to higher-grade cervical lesions [13]. In addition, the greater the extent of cervical lesions, the greater the possibility of disease progression and occult invasion [14]. Priscila even more concretizes the calculation of cervical lesion area, and they considered that the lesion area greater than the threshold of 30,337.02 pixels was related to the occurrence of HSIL and invasive cancer [14]. The authors found that 22 patients (88%) had lesion range ≥ 3 quadrants among those patients with postoperative pathological upgrading. There was also a correlation between the size of cervical lesions and postoperative pathological upgrading. Therefore, patients with HSIL should be alert to the risk of invasive cancer when the lesion involved glands or the lesion range ≥ 3 quadrants.

HPV16/18 infection and TCT ≥ ASC-H were also risk factors for pathological upgrading after total hysterectomy directly for HSIL. There were so many studies shown that the occurrence of cervical cancer is related to persistent infection of HPV16/18. Among the 25 patients with postoperative pathological invasive cancer, the average age was 63 years old, and 76% patients had HPV16/18 infection. The reason may be related to the decreased hormone level in elderly women, the decreased immune function of the body, the weakened natural clearance of HPV, and the increased risk of HPV persistent infection [15]. In this study, compared with those without HPV16/18 infection, patients with HPV16/18 infection had a 5.382 times increased risk of postoperative lesion escalation. Terresa reported that HPV16/18 infection accounted for about 70% of global cervical cancer patients [16] and Jae-Eun Lee reported that HPV-negative cervical cancer only accounted for 3–8% [17]. Meanwhile, we found that 56.0% (14/25) of the patients were TCT ≥ ASC-H. Ciavattini found that the proportion of TCT ≥ ASCH in direct hysterectomy of HSIL was higher (84.3%) [18], and the higher the level of cervical cytology examination, the higher the risk of cervical invasive carcinoma [19]. Therefore, patients with HPV16/18 infection and TCT ≥ ASCH need to be paid enough attention and actively treated to reduce the risk of cancer.

In this study, 25 patients with postoperative pathological upgrading underwent extrafascial hysterectomy for the first time, all of which are early cervical cancer. Up to now, we have found no recurrence or death cases, and the median follow-up period was 21 months (range 2–59 months). It is suggested that only extrafascial total hysterectomy for early cervical cancer may also achieve good survival benefits. Vandré also found that the 3-year disease-free survival(DFS) rate of patients with early cervical cancer after simple hysterectomy was 95%, the 3-year DFS rate after modified radical hysterectomy was 100%, and the 5-year overall survival(OS) rate was 90% and 91%, respectively, and it showed that early-stage cervical cancer can also obtain good survival rate after simple hysterectomy [20]. The SHAPE study at the annual meeting of the American Society of Clinical Oncology(ASCO) in 2023 pointed out that the effect of simple total hysterectomy in the treatment of early cervical cancer was not inferior to that of radical hysterectomy, and there was no significant statistical difference in recurrence-free survival(RFS) rate and OS rate [21]. Marie found that simple hysterectomy(SH) was non inferior to radical hysterectomy(RH) with respect to 3-year pelvic recurrence among women with low-risk cervical cancer [22]. Another meta-analysis showed that there was no significant difference in recurrence rate and overall survival in patients with stage IA2-IB1 cervical cancer who received SH or RH, while the SH group had better surgery-related outcomes [23]. The results indicate that simple hysterectomy also can achieve a good survival benefits for early-stage cervical cancer. Of course, this still needs to be confirmed by more high-quality prospective studies. Simple total hysterectomy may be a more appropriate surgical method for patients with early cervical cancer who meet the treatment criteria.

We analyzed the demographic and clinicopathological characteristics of these 314 patients, and found that most of the patients were low education (88.5%) and low income (77.1%) groups. The possible reasons for their tendency to choose direct hysterectomy are fear of cancerous diseases and economic pressure of secondary surgery. Strengthening health education for patients can reduce their cancer-phobia and improve their treatment compliance. In addition, we also found that 73.2% of the patients came from primary hospitals, which may be related to the non-standard diagnosis and treatment in primary hospitals and the level of diagnosis and treatment of doctors. Therefore, it is necessary to strengthen the training of doctors in primary hospitals and study the guidelines of expert consensus.

### Limitation

Our study has several limitations. ①. This study is a retrospective study with inherent design bias; ②. The follow-up time in this study was short; ③. Not all patients underwent preoperative examinations, such as ECC, SCCA, or pelvic MRI, as recommended by the guidelines; ④. Recurrent HPV infection, which may be associated with the progression of cervical cancer, was not included in the discussion of this study; ⑤. CIN2 and CIN3 were not strictly distinguished,which may lead to a bias. Despite these limitations, this study included data from a sample of 5 hospitals. Therefore, our findings provide guidance for clinical decision making and further research.

## Conclusion

In conclusion, patients with HSIL have a risk of occult cervical cancer. Lesions involved glands, Lesion range ≥ 3 quadrants, HPV 16/18 infection, TCT ≥ ASC-H were independent risk factors for HSIL combined with occult cervical cancer. The prognosis of biopsy-proved HSIL patients who underwent extrafascial hysterectomy and unexpected early invasive cancer was later identified on specimen may be good.

### Supplementary Information

Supplementary Material 1.

## Data Availability

The datasets generated and analysed during the current study are not publicly available due avoid unreasonable use by third parties or organizations, but are available from the corresponding author on reasonable request.

## Abbreviations

CKC: Cold knife conzation
LEEP: Loop electrosurgical excision procedure
HSIL: High-grade squamous intraepithelial lesion
CAP: American Society for Pathology
ASCCP: American Society for Colposcopy and Cervical Pathology
CIN: Cervical intraepithelial neoplasia
HR-HPV: High-risk human papillomavirus
TCT: Thincytologic test
ROC: Receiver operator characteristic
AUC: Area under receiver operator characteristic curve
NILM: Negative for intraepithelial lesion or malignancy
LSIL: Low grade cervical intraepithelial lesions
HSIL: High grade cervical intraepithelial lesions
ASCUS: Atypical squamous cells of undetermined significance
ASC-H: Atypical squamous cells, cannot exclude high-grade squamous intraepithelial lesion
AGC: Atypical glandular cells
