# Supplementary material for: Clinical analysis of 314 patients with high-grade squamous intraepithelial lesion who underwent total hysterectomy directly: a multi-center, retrospective cohort study
Source: BMC Cancer. 2024 May 9;24:575. doi: 10.1186/s12885-024-12342-2 (PMC11080298; doi:10.1186/s12885-024-12342-2)
Supplement: Supplementary file 1 — Supplementary Material 1. [file 12885_2024_12342_MOESM1_ESM.docx]

**characteristics of 25 patients pathological upgrading (cancer) after extrafascial hysterectomy**

| Number | Age | Menopausal status | TZ | Glandular involvement | HPV | TCT | Lesion range | Cancer staging | Surgical methods | Adjuvant Therapy | Follow-up time（month） |
| --- | --- | --- | --- | --- | --- | --- | --- | --- | --- | --- | --- |
| 1 | 70 | Yes | 3 | Yes | 16 | ASC-US | 4 | IB1 | Extrafascial hysterectomy + Lymph node dissection | Radiotherapy | 13 |
| 2 | 54 | Yes | 3 | Yes | 18 | HSIL | 2 | IB1 | Extrafascial hysterectomy + Lymph node dissection | Chemotherapy | 30 |
| 3 | 71 | Yes | 3 | Yes | 16、18、52 | HSIL | 3 | IA2 | Extrafascial hysterectomy | - | 7 |
| 4 | 58 | Yes | 3 | Yes | 16 | ASC-US | 4 | IA2 | Extrafascial hysterectomy | Radiotherapy + Chemotherapy | 17 |
| 5 | 58 | Yes | 3 | Yes | 16 | ASC-US | 2 | IB1 | Extrafascial hysterectomy | Radiotherapy + Chemotherapy | 21 |
| 6 | 59 | Yes | 3 | Yes | 16 | NILM | 2 | IA1 | Extrafascial hysterectomy + Lymph node dissection | - | 2 |
| 7 | 77 | Yes | 3 | Yes | Negative | ASC-US | 4 | IB1 | Extrafascial hysterectomy + Lymph node dissection | - | 36 |
| 8 | 71 | Yes | 3 | No | 16、33、58 | HSIL | 3 | IA2 | Extrafascial hysterectomy + Lymph node dissection | Radiotherapy | 31 |
| 9 | 60 | Yes | 3 | Yes | 16、81 | ASC-US | 3 | IA2 | Extrafascial hysterectomy | Radiotherapy | 9 |
| 10 | 59 | Yes | 2 | Yes | 16 | ASC-H | 4 | IA2 | Extrafascial hysterectomy | - | 29 |
| 11 | 56 | Yes | 2 | Yes | 16 | ASC-H | 4 | IB1 | Extrafascial hysterectomy | - | 30 |
| 12 | 58 | Yes | 3 | No | 82 | ASC-US | 4 | IA1 | Extrafascial hysterectomy | - | 37 |
| 13 | 74 | Yes | 3 | No | 16、53、58 | HSIL | 4 | IB1 | Extrafascial hysterectomy | - | 44 |
| 14 | 67 | Yes | 3 | No | 58 | ASC-US | 3 | IB1 | Extrafascial hysterectomy | - | 41 |
| 15 | 43 | No | 3 | Yes | 16、18 | ASC-H | 2 | IB1 | Extrafascial hysterectomy | - | 59 |
| 16 | 66 | Yes | 3 | Yes | 33 | ASC-H | 4 | IA1 | Extrafascial hysterectomy | - | 57 |
| 17 | 65 | Yes | 3 | Yes | 16、18 | ASC-US | 4 | IA1 | Extrafascial hysterectomy | - | 55 |
| 18 | 44 | No | 3 | Yes | 16、59 | ASC-H | 4 | IA1 | Extrafascial hysterectomy | - | 21 |
| 19 | 61 | Yes | 3 | Yes | 33、39、52 | HSIL | 4 | IA1 | Extrafascial hysterectomy | - | 16 |
| 20 | 76 | Yes | 3 | Yes | 16、56、58、68、42 | ASC-US | 4 | IA1 | Extrafascial hysterectomy | - | 11 |
| 21 | 75 | Yes | 3 | Yes | 16、35、51、52、58、66 | ASC-US | 4 | IA1 | Extrafascial hysterectomy | - | 3 |
| 22 | 64 | Yes | 3 | Yes | 16 | HSIL | 3 | IB1 | Extrafascial hysterectomy | - | 3 |
| 23 | 62 | Yes | 3 | Yes | 33、16 | ASC-H | 3 | IA1 | Extrafascial hysterectomy | - | 4 |
| 24 | 73 | Yes | 3 | Yes | 6、16、33、58、68 | ACG | 4 | IA2 | Extrafascial hysterectomy | Chemotherapy | 10 |
| 25 | 55 | Yes | 3 | Yes | Negative | HSIL | 3 | IB1 | Extrafascial hysterectomy | Chemotherapy | 6 |
